# Early Pleistocene origin and extensive intra-species diversity of the extinct cave lion

**DOI:** 10.1038/s41598-020-69474-1

**Published:** 2020-07-28

**Authors:** David W. G. Stanton, Federica Alberti, Valery Plotnikov, Semyon Androsov, Semyon Grigoriev, Sergey Fedorov, Pavel Kosintsev, Doris Nagel, Sergey Vartanyan, Ian Barnes, Ross Barnett, Erik Ersmark, Doris Döppes, Mietje Germonpré, Michael Hofreiter, Wilfried Rosendahl, Pontus Skoglund, Love Dalén

**Affiliations:** 1 Centre for Palaeogenetics, Svante Arrhenius väg 20C, 106 91 Stockholm, Sweden; 2 Department of Bioinformatics and Genetics, Swedish Museum of Natural History, Stockholm, Sweden; 3 Institute for Biochemistry and Biology, University of Potsdam, Karl-Liebknecht-Str. 24-25, 14476 Potsdam, Germany; 4 Reiss-Engelhorn-Museen, Zeughaus C5, 68159 Mannheim, Germany; 5 Academy of Sciences of Sakha Republic, Lenin Avenue 33, Yakutsk, Sakha Republic (Yakutia) Russia; 6 Museum “Severnyi Mir”, Yakutsk, Sakha Republic (Yakutia) Russia; 7 Mammoth Museum of Institute of Applied Ecology of the North, North-Eastern Federal University, Yakutsk, Sakha Republic (Yakutia) Russia; 8 Institute of Plant and Animal Ecology, Russian Academy of Sciences, 202 Marta 8 St., Ekaterinburg, Russia 620144; 9 Department of Paleontology, University of Vienna, Althanstrasse 14, 1090 Vienna, Austria; 10 North-East Interdisciplinary Scientific Research Institute n.a. N.A. Shilo FEB RAS (NEISRI FEB RAS), Portovaya Str., 16, Magadan, Russia 685000; 11 Department of Earth Sciences, Natural History Museum, London, UK; 12 Natural History Museum of Denmark, University of Copenhagen, Copenhagen, Denmark; 13 OD Earth and History of Life, Royal Belgian Institute of Natural Sciences, Vautierstraat 29, 1000 Brussel, Belgium; 14 The Francis Crick Institute, 1 Midland Road, London, NW1 1AT UK; 15 Department of Zoology, Stockholm University, Stockholm, Sweden

**Keywords:** Evolutionary genetics, Zoology

## Abstract

The cave lion is an extinct felid that was widespread across the Holarctic throughout the Late Pleistocene. Its closest extant relative is the lion (*Panthera leo*), but the timing of the divergence between these two taxa, as well as their taxonomic ranking are contentious. In this study we analyse 31 mitochondrial genome sequences from cave lion individuals that, through a combination of ^14^C and genetic tip dating, are estimated to be from dates extending well into the mid-Pleistocene. We identified two deeply diverged and well-supported reciprocally monophyletic mitogenome clades in the cave lion, and an additional third distinct lineage represented by a single individual. One of these clades was restricted to Beringia while the other was prevalent across western Eurasia. These observed clade distributions are in line with previous observations that Beringian and European cave lions were morphologically distinct. The divergence dates for these lineages are estimated to be far older than those between extant lions subspecies. By combining our radiocarbon tip-dates with a split time prior that takes into account the most up-to-date fossil stem calibrations, we estimated the mitochondrial DNA divergence between cave lions and lions to be 1.85 Million ya (95% 0.52– 2.91 Mya). Taken together, these results support previous hypotheses that cave lions existed as at least two subspecies during the Pleistocene, and that lions and cave lions were distinct species.

## Introduction

The cave lion (*Panthera spelaea*) was an apex predator across the Holarctic^1,2^ until their extinction at the end of the Pleistocene^3^ (last occurrence in the fossil record 14,219 ± 112 cal BP^4^). Cave lions were larger than extant lions^5^, and Pleistocene cave art suggests that they did not have manes. However they may have shared several behavioural traits with their modern counterparts, such as group living and courtship rituals^6^.

Cave lion taxonomy has been contentious, being variously considered a subspecies of *Panthera leo*^7,8^, a sister species to extant lions (*Panthera spelaea*)^5,9^, or even being more closely related to the tiger (*Panthera tigris spelaea*)^10^. In particular, the molecular estimate of the timing of the split between cave lions and extant lions has varied considerably between studies (~ 600 kya^8^; 1.23–2.93 mya^11^). Ersmark et al.^12^ identified two major cave lion mitochondrial DNA haplogroups (based on ~ 348 bp of ATP8 and control region sequences) and showed that there was an association between the age of the specimen and its haplogroup, with one of the two haplogroups disappearing ~ 41 kya. Morphological analysis of skulls and mandibles has shown that cave lions from Yakutia, Alaska and the Yukon Territory are smaller than those from Europe, and led to the conclusion that Beringian lions should be recognised as a distinct subspecies “*Panthera spelaea vereshchagini* n.subsp"^13^. However, because all previous genetic studies of cave lions have either used only a small mitochondrial fragment or relied on limited sample number, phylogenetic structure within cave lions, and between cave lions and extant lions has remained largely unresolved.

In this study, we investigated mitochondrial genome diversity in 31 cave lions from across their entire prehistoric range, and from an even temporal spread between the last occurrence in the fossil record to beyond the limit of radiocarbon dating. We also generated multiple new radiocarbon dates, allowing us to (1) Use genetic data to estimate the age of specimens with ambiguous or infinite radiocarbon dates, (2) Estimate the date of the split between *P. leo* and *P. spelaea*, and (3) Investigate intra-species mitochondrial diversity across the entire historical distribution of cave lions.

## Results/discussion

**Figure 1:**
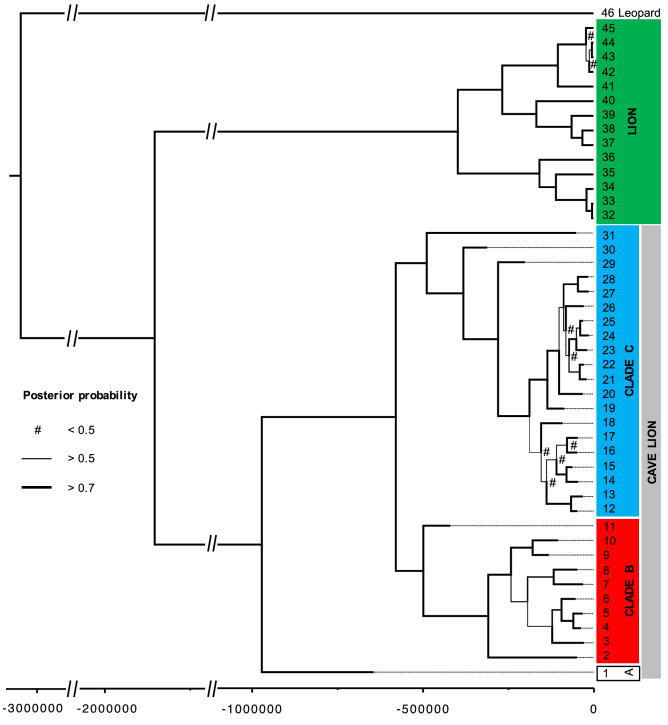
Phylogeny based on 7,929 bp of the mitochondrial genome. Priors used for the TMRCA split between lions and cave lions were from Barnett et al.^11^ (2.08 mya, standard deviation of 0.52 mya). Branch thickness corresponds to posterior support (values given in Figure S5), with any values less than 0.5 shown by a hash). The eleven oldest splits all have posterior support > 0.95. Tip numbers correspond to sample IDs in Table S1, the x-axis scale is in ^14^C years before present.

Mitochondrial genome sequences show that cave lions and modern lions fall into two well-supported reciprocally monophyletic clades (Fig. 1; posterior = 1.00). New radiocarbon dates for the cave lions range between 28.0 kya (thousand radiocarbon years before present; ± 110 years) to beyond the radiocarbon limit (Table S1). By combining these ^14^C dates as tip priors, alongside a TMRCA prior that takes into account fossil calibration, we estimate this divergence between the cave lions and lion clades to be 1.85 mya (million radiocarbon years before present; 95% credibility interval: 0.52–2.91 mya). This date is in agreement with the previous estimate by Barnett et al.^11^ (1.23–2.93 million years) that incorporate fossil calibrations, rather than only molecular estimates (1 kb of the Cytochrome b gene from two cave lion individuals, ~ 600 kya^8^; whole genome data from modern lions and two cave lion individuals, ~ 500 kya^14^). Using only ^14^C tip dates to inform the analysis leads to a divergence estimate that is closer to the younger molecular estimates, at 550 kya (0.17–3.96 mya; Figure S2). It is expected that tip dates will often give younger divergence date estimates than fossil calibrations, due to time dependency of molecular rates^15^. In the present study we judge the older date estimate (that combines the ^14^C tip date and divergence date priors) to be more relevant because, 1. The posterior estimate is based on more prior information, 2. The credibility intervals are very wide when using only the tip dates, and encompass the credibility intervals from the combined approach, 3. Tip date randomization shows that the tip dates we are using as priors are providing reliable mutation rate information into the tree (Figure S3), and 4. It is important to use calibration points that are as close as possible to the date being estimated^15^, and in the present study we are focussing on the evolutionary processes that occur throughout the time period between the cave lion / lion divergence until the terminal tips. For the present study we therefore use the phylogeny with the older split date estimate. However, it should be noted that, 1. This estimate relies heavily on fossil calibrations, and would need to be reconsidered if these were revised in the future^11^, and 2. The lower end of the credibility intervals for our divergence estimate is 520 kya and so previous younger divergence estimates based only on molecular data are still compatible with our findings. In addition, the extensive dataset of ^14^C calibration points and mitochondrial genomes presented here is the first such dataset within Felidae, and the younger divergence (and therefore faster mutation rate) estimate may be more applicable for future within-species population studies on other felids.

Most of the nodes within the cave lion clade also have strong posterior support (the eight deepest splits have posterior support ≥ 0.99). The oldest of these splits (0.97 mya; 95% credibility interval 0.20–1.61 mya) separates a single specimen (from Barnett et al.^11^; Genbank accession number: KX258452; “Lineage A”) from the rest of the specimens, and molecular tip dating estimates it to be 643 kya (95% credibility interval: 211 kya—1.00 mya). The specimen is from the remains of a cave lion found in Bilibino, Russia in 2008^16^. The find included an incomplete postcranial skeleton (67 elements) and some red hair that returned a radiocarbon date different to that of the bone (bone: > 61.0 kya, hair: 28.7 kya [± 130]) and so may have been from a different individual. However, the authors concluded that it was likely that contamination had affected the radiocarbon date and it was actually much older. It was the mitochondrial genome sequence (from Barnett et al.^11^) generated from this hair that was used in the present study. If this sequence is genuine, this specimen therefore represents the only currently sequenced example of a highly distinct cave lion mitochondrial lineage where even the lowest molecular date estimate is considerably (a factor of 2.2) older than the radiocarbon result reported in Kirillova et al.^16^, suggesting that that date has indeed been affected by contamination. However, considering the uncertain provenance of this sample, its unique placement on the phylogeny, and because the raw sequencing reads that were used to generate the sequence are not available to validate the consensus, conclusions based on the distinctiveness of this specimen should be treated with caution. Future genetic work on one of the bone elements from the skeleton would be very valuable, in order to confirm the original sequence from the hair, and to determine if it is from the same individual as the skeleton.

**Figure 2:**
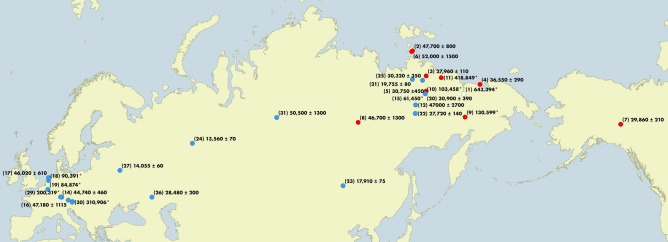
Location of all specimens included in this study (see Table S1 for detailed sample information). Red and blue dots correspond to clades B and C respectively, and the white dot corresponds to the single representative of “Lineage A” (Fig. 1) Numbers correspond to sample number (in parentheses) and radiocarbon years before present, unless suffixed by an asterisk, in which case they were estimated by BEAST (see Methods; 95% credibility intervals given in Table S2). Map created using QGIS v2.12.1-Lyon (https://qgis.org/en/site/).

The second deepest split (578 kya; 95% credibility interval 124 kya–1.08 mya) then partitions the remaining haplotypes approximately evenly (“Clade B & C”). Clade B contained specimens dated at between 28.0 kya (± 110 years, ^14^C dated) and 419 kya (91.8–827 kya, molecular dating). Clade C contained specimens dated between 13.6 kya (± 70 years; the youngest cave lion specimen ever found is 12.4 kya [± 50 years]^4^) to 311 kya (64.5–665 kya). Interestingly, we identified a strong association between mitochondrial lineage and geography (Fig. 2). While there was some spatial overlap between the clades B & C, clade B was almost entirely restricted to Beringia with all but one sample restricted to the east of the Yana River (as far as Quartz Creek, Yukon Territory, Canada to the East; Fig. 2). Clade C occurred throughout Eurasia, from as far west as the Netherlands (North Sea, 5.0°E), but did not extend into Beringia (Fig. 2; excluding the specimen from eastern Beringia, 139.3°W). We were able to incorporate the two mitochondrial genomes from Barnett et al.^9^, however once we had excluded the *numt regions from our alignment* there was not sufficient overlap to be able to include the sequences from Ersmark et al.^12^ in the present study as a comparison. Ersmark et al.^12^ and Barnett et al.^9^ both identified an association between cave lion specimen age and haplogroup: They identified two main haplogroups, one of which they did not detect in any samples younger than c. 37 kya. They also noted that all haplotypes observed in samples from regions outside of Beringia that are younger than 41 kya belonged exclusively to only one of their haplogroups. While neither of the two main lineages we identified died out at ~ 41 kya (Figure S4) as suggested by Ersmark et al.^12^, all specimens younger than 28 kya belonged to lineage C, suggesting that one of the major cave lion mitogenome lineages may indeed have gone extinct as much as 10,000 years before the species extinction.

Our results suggest that cave lions diverged from present-day lions early during the Pleistocene, a period that appears to be important in the diversification of a number of other megafaunal species (e.g. cave bears from their sister clade, the brown and polar bears, 1.59 mya^17^; the main mammoth clades, ~ 2.0–1.0 mya^18^; the split between African and Eurasian hyena populations, ~ 2.5 mya,^19^). This result is in line with previous studies that have hypothesised that cave lions and modern lions are distinct species, based on morphological^5^ and genetic data^9^. Within the cave lion, we identified two mitochondrial clades that diverged approximately c. 578 kya, and a lineage represented by a single individual that diverged c. 971 kya, all of which ultimately went extinct before the start of the Holocene. Both these splits are considerably older than the one between the extant lion subspecies (Supplementary Results). The divergence times between the two cave lion clades, and tip dates within them, suggest that these clades separated during the mid-Pleistocene and appear to have had distinct distributions since that time. This geographical distribution of the two cave lion clades (Fig. 2) is consistent with previous findings that cave lion skulls and mandibles from Beringia (Yakutia, Alaska and Yukon Territory) are significantly smaller than those from Europe^13^. In addition, it is likely that Beringian and European cave lions had different prey preferences, with the former focussing on bison and horses, and the latter on reindeer^1,16^. The genetic data therefore supports previous hypotheses, based on morphology and ecology, that the Beringian cave lion was a separate subspecies (*Panthera spelaea vereshchagini*)^13^. These results therefore provide the first complete description of the evolutionary genetic history of what used to be Europe’s most widespread mega-carnivore, across its entire Holarctic distribution, from the time of its divergence until its ultimate extinction.

## Methods

### Samples and DNA extraction

Fifty-nine cave lion bone, teeth and skin samples were collected from a variety of locations across the Holarctic (see Fig. 1; details of samples used in the final dataset are given in Table S1).

For bone and teeth samples, the outside surface was cleaned with 0.5% bleach and then a thin layer was removed using a Dremel drill. Approximately 50–100 mg bone powder was then taken from the newly exposed part of the sample. For a subset of the samples (“sample set B”, non-permafrost and no previous DNA sequencing success, but with sufficient bone material for the below methods, n = 8) we used the sampling procedure described in Alberti et al.^20^ to drill bone powder. The bone powder for all samples was digested overnight in 1 ml of extraction buffer (0.25 mg/ml Proteinase K, 0.05% Tween 20, 0.45 M EDTA [pH 8.0]), and DNA extracted using the methodology of Dabney et al.^21^. For the three skin samples (“sample set C”, the remaining 48 samples are referred to as “sample set A”, Table S1 [only samples that were used in the final analysis are included in this table]), we instead digested the tissue in a buffer optimised to digest keratin-rich tissues after Gilbert et al.^22^, before extracting DNA using the methodology of Yang et al.^23^.

### Library preparation, sequencing and radiocarbon dating

Sample set A and C: We built double-stranded Illumina libraries according to Meyer and Kircher^24^. Specifically, we used 20 μl of DNA extract in a 40 μl blunt-end repair reaction with a final concentration of 1 × buffer Tango, 100 μM of each dNTP, 1 mM ATP, 20 U T4 polynucleotide kinase (Thermo Scientific) and 3U USER enzyme (New England Biolabs, to excise uracil residues resulting from post-mortem damage). Samples were incubated for 3 h at 37 °C, followed by the addition of 4 U T4 DNA polymerase (Thermo Scientific) and incubation at 25 °C for 15 min and 12 °C for 5 min. The reaction was cleaned using MinElute spin columns following the manufacturer's protocol and eluted in 20ul EB Buffer. We then performed an adapter ligation step where DNA fragments of the library were ligated to IS1 and IS3 adapters. This reaction was performed in a 40 μl reaction volume using 20 μl of blunted DNA from the clean-up step, 20 pmol of each adapter, 1 × T4 DNA ligase buffer, 5% PEG-4000 and 5U T4 DNA ligase (Thermo Scientific). Sample were incubated for 30 min at room temperature and cleaned again using MinElute spin columns following the manufacturer's protocol. Next, we performed an adapter fill-in reaction in 40 μl final volume using 20 μl adapter ligated DNA with a final concentration of 1 × Thermopol Reaction Buffer, 250 μM of each dNTP, and 12 U *Bst* Polymerase, Long Fragment. The library was incubated at 37 °C for 20 min, and heat-inactivated at 80 °C for 20 min. This library was then used as stock for two indexing PCR amplifications using double-unique p5-p7 indexed primers. The first amplification was performed in a volume of 25 μl with 3 μl of adapter-ligated library as template, with the following final concentrations: 1 × AccuPrime reaction mix, 0.3 μM P7 + p5 indexing primer mix, 1.25 U AccuPrime Pfx (Thermo Scientific) and the following cycling protocol: 95 °C for 2 min, 12 cycles at 95 °C for 30 s, 55 °C for 30 s, 72 °C for 1 min and a final extension at 72 °C for 5 min. The second PCR amplification was then carried out with the same reaction conditions, but adjusting the number of cycles up to 14, or down to 9, depending on the relative brightness of the PCR product on a 2% agarose gel. Amplified libraries were then pooled in approximate equimolar amounts. This was done using a linear regression between our gel-based molarity estimates and a subset of PCR products run on a high-sensitivity DNAchip on a Bioanalyzer 2,100 (Agilent, Santa Clara, CA, USA), to adjust the remaining gel-based estimates.

Purification and size selection of the pooled libraries was performed using Agencourt AMPure XP beads (Beckman Coulter, Brea, CA, USA), using a 0.5X and 1.8X to 1.6 × bead:DNA ratio to remove long and short fragments, respectively, and then re-measured on a Bioanalyzer. One PCR reaction for each of the libraries was sequenced on an Illumina NovaSeq6000 S4 (150 bp paired-end [PE] setup) at the Sci*Life*Lab sequencing facility in Stockholm (for a total of 1.50e^9^ PE reads, mean of 2.96e^7^ PE per sample). Based on preliminary results, samples with a low number of mitochondrial reads were excluded from further analysis and nine samples with an intermediate number of mitochondrial reads underwent additional sequencing (two new PCRs and index pairs; 1.08e^9^ additional PE reads total, mean of 1.21e^8^ PE per sample) to gain sufficient reads for obtaining mitochondrial genomes.

Sample set B: Library built was carried out following the single stranded approach described in Gansauge et al.^25^ with an additional pre-treatment of the extract with 0.5ul of USER enzyme for 15 min at 37C (modified from Meyer et al.^26^). The optimal number of library amplification PCR cycles were determined using qPCR as described in Gansauge and Meyer^27^. Indexing PCR was performed in a final reaction volume of 80 ul: 20 ul template library, 1 × Accuprime Pfx reaction mix, 0.025 U/ul Accuprime Pxf polymerase and Illumina P5-P7 primers to generate dual-indexed library molecules. The final libraries were pooled together in equimolar amounts according to their concentration and length distribuition determined respectively with Qubit 2.0 and 2,200 Tapestation (Agilent Technologies). Libraries were then sequenced on the Illumina NextSeq 500 sequencing platform producing 75-bp single-end reads using custom primers as described in Paijmans et al.^28^. Given the low endogenous DNA yielded, two rounds of mitochondrial enrichment were performed for each of these libraries. The hybridization capture was performed using myBaits custom kit (Arbor Biosciences) with a designed RNA bait-set targeting several mammals mitochondrial genomes including the modern lion (*Panthera leo*, KF776494). The capture procedure was carried out as described in the myBaits manual v.4 (https://arborbiosci.com/wp-content/uploads/2019/08/myBaits-Manual-v4.pdf) with the following settings for the hybridiyation step: 65 °C for 48 h.

### Final dataset

The above steps lead to a final dataset of 31 samples with sufficient coverage to call mitochondrial genomes (with at least 80% of the sequence at > 3X coverage, mitogenome coverage ranged between 4.8X and 900X). Of these, 14 have been included in a previous study (~ 348 bp of ATP8 and control region sequences^12^), and two are full mitochondrial sequences that had previously been published (genbank: KX258451 & KX258452)^11^. The remaining 15 specimens have not undergone any DNA analysis before. Twelve of these specimens were radiocarbon dated at The Oxford Radiocarbon Accelerator Unit, UK. Sequencing reads from all samples were mapped to the cave lion mitochondrial genome (genbank accession number KX258452), and duplicates were removed using a custom perl script that removes reads with identical start and end positions, keeping the first observed such read. It has been known for some time that the *Panthera* genus has undergone a large translocation of mtDNA into the nuclear genome (*numt*)^29^. We identified the extent of this *numt* region based on variations in coverage (Supplementary Methods), and trimmed it from all individuals for subsequent analyses, leaving a final trimmed sequence of 7,929 bp.

### Molecular dating

Nine individuals had unknown dates (five greater than the ^14^C limit, three undated, and one not dated by us with an ambiguous date, see Kirillova et al.^16^). We therefore attempted to date them molecularly by treating the dates for those tips in the tree as a prior with a normal distribution, 97.5% of which was greater than the minimum date given by the radiocarbon dating (see Table S2).

### Mitochondrial genome phylogeny

Mitochondrial phylogenies were run in BEAST v1.10.1^30^, using the full mitochondrial sequence, trimmed for the *numt* region (trimmed sequence: 7,929 bp). We used an HKY + I substitution model (highest BIC and DT support in JModelTest2^31^), uncorrelated relaxed lognormal clock and coalescent constant size tree prior. We combined 12 MCMC chains (each run for 200 M iterations), after excluding the first 25% of values as a burnin. We included mitochondrial genomes from representatives of the major extant lion lineages, and leopard as an outgroup (*Panthera pardus*; Genbank accession: KP001507). One tree was created with a TMRCA (time to most recent common ancestor) prior from Barnett et al. (2016; a normal distribution with a mean of 2.08 mya, and a standard deviation of 517 mya [95% CI’s = 1.23–2.93 mya]; Fig. 1) to take into account the prior expectation based on fossil calibration, and one was created with no prior for TMRCA for cave lions and lions. In order to investigate if the tip dates we are using as priors are providing reliable mutation rate information into the tree, we carried out a randomization approach, whereby prior tip dates were randomly assigned (12 replicates) to the cave lion tips to investigate how it affects the molecular clock estimate^32^. We ran each replicate for 200 M iterations and kept all other parameters the same (as the tree with no TMRCA prior).

### Ethics statement

No living animals were used in this study, and any samples used in this study that may have been the result of cave lions being euthanized would have been outside the authors’ control and remit. The samples were obtained with permission from all sample providers.

## Supplementary information

Supplementary Information.

Supplementary Table 1.

Supplementary Table 2.

## References

[1] Bocherens H (2011). Isotopic evidence for dietary ecology of cave lion (*Panthera spelaea*) in North-Western Europe: prey choice, competition and implications for extinction. Quat. Int..

[2] Antón M, Galobart A, Turner A (2005). Co-existence of scimitar-toothed cats, lions and hominins in the European Pleistocene. Implications of the post-cranial anatomy of Homotherium latidens (Owen) for comparative palaeoecology. Quat. Sci. Rev..

[3] Barnosky AD, Koch PL, Feranec RS, Wing SL, Shabel AB (2004). Assessing the causes of late pleistocene extinctions on the continents. Science.

[4] Stuart AJ, Lister AM (2007). Patterns of late quaternary megafaunal extinctions in Europe and northern Asia. CFS Cour. Forschungsinstitut Senckenb..

[5] Sotnikova M, Nikolskiy P (2006). Systematic position of the cave lion *Panthera spelaea* (Goldfuss) based on cranial and dental characters. Quat. Int..

[6] Packer C, Clottes J (2000). When lions ruled france. Nat. Hist..

[7] Kurtén B (1985). The Pleistocene lion of Beringia. Ann. Zool. Fenn..

[8] Burger J (2004). Molecular phylogeny of the extinct cave lion Panthera leo spelaea. Mol. Phylogenet. Evol..

[9] Barnett R (2009). Phylogeography of lions (*Panthera leo ssp*.) reveals three distinct taxa and a late Pleistocene reduction in genetic diversity. Mol. Ecol..

[10] Der Groiss JT (1996). Höhlentiger *Panthera tigris spelaea* (Goldfuss). Neues Jahrb. für Geol. und Paläontologie - Monatshefte..

[11] Barnett R (2016). Mitogenomics of the extinct cave lion, Panthera spelaea (Goldfuss, 1810), resolve its position within the panthera cats. Open Quat..

[12] Ersmark E (2015). Population demography and genetic diversity in the pleistocene cave lion. Open Quat..

[13] Baryshnikov G, Boeskorov G (2001). The pleistocene cave lion, *Panthera spelaea* (Carnivora, Felidae) from Yakutia, Russia. Cranium.

[14] de Manuel M (2020). The evolutionary history of extinct and living lions. Proc. Natl. Acad. Sci. USA.

[15] Ho SYW, Larson G (2006). Molecular clocks: When times are a-changin’. Trends Genet..

[16] Kirillova IV (2015). On the discovery of a cave lion from the Malyi Anyui River (Chukotka, Russia). Quat. Sci. Rev..

[17] Barlow A (2020). Middle pleistocene cave bear genome calibrates the evolutionary history of palaearctic bears. SSRN Electron. J..

[18] Chang D (2017). The evolutionary and phylogeographic history of woolly mammoths: a comprehensive mitogenomic analysis. Sci. Rep..

[19] Westbury MV (2020). Hyena paleogenomes reveal a complex evolutionary history of cross-continental gene flow between spotted and cave hyena. Sci. Adv..

[20] Alberti F (2018). Optimized DNA sampling of ancient bones using Computed Tomography scans. Mol. Ecol. Resour..

[21] Dabney J (2013). Complete mitochondrial genome sequence of a Middle Pleistocene cave bear reconstructed from ultrashort DNA fragments. Proc. Natl. Acad. Sci..

[22] Gilbert MTP (2007). Whole-genome shotgun sequencing of mitochondria from ancient hair shafts. Science.

[23] Yang DY, Eng B, Waye JS, Dudar JC, Saunders SR (1998). Improved DNA extraction from ancient bones using silica-based spin columns. Am. J. Phys. Anthropol..

[24] Meyer M, Kircher M (2010). Illumina sequencing library preparation for highly multiplexed target capture and sequencing. Cold Spring Harb. Protoc..

[25] Gansauge MT (2017). Single-stranded DNA library preparation from highly degraded DNA using T4 DNA ligase. Nucl. Acids Res..

[26] Meyer M (2012). A high-coverage genome sequence from an archaic Denisovan individual. Science.

[27] Gansauge MT, Meyer M (2013). Single-stranded DNA library preparation for the sequencing of ancient or damaged DNA. Nat. Protoc..

[28] Paijmans, J. L. A. *et al.* Sequencing single-stranded libraries on the Illumina NextSeq 500 platform. arXiv:1711.11004 (2017).

[29] Kim JH (2006). Evolutionary analysis of a large mtDNA translocation (numt) into the nuclear genome of the Panthera genus species. Gene.

[30] Suchard MA (2018). Bayesian phylogenetic and phylodynamic data integration using BEAST 1.10. Virus Evol..

[31] Darriba D, Taboada GL, Doallo R, Posada D (2012). jModelTest 2: more models, new heuristics and parallel computing. Nat. Methods.

[32] Ho SYW (2011). Bayesian estimation of substitution rates from ancient DNA sequences with low information content. Syst. Biol..

