# Supplementary material for: Early Pleistocene origin and extensive intra-species diversity of the extinct cave lion
Source: Sci Rep. 2020 Jul 28;10:12621. doi: 10.1038/s41598-020-69474-1 (PMC7387438; doi:10.1038/s41598-020-69474-1)
Supplement: Supplementary file 1 — Supplementary Information. [file 41598_2020_69474_MOESM1_ESM.docx]

**SUPPLEMENTARY: Early-Pleistocene origin and extensive intra-species diversity of the extinct cave lion**

David W. G. Stanton^1,2^, Federica Alberti^3,4^, Valery Plotnikov^5^, Semyon Androsov^6^, Semyon Grigoriev^7,†^, Sergey Fedorov^7^, Pavel Kosintsev^8^, Doris Nagel^9^, Sergey Vartanyan^10^, Ian Barnes^11^, Ross Barnett^12^, Erik Ersmark^1,2^, Doris Döppes^4^, Mietje Germonpré^13^, Michael Hofreiter^3^, Wilfried Rosendahl^4^, Pontus Skoglund^14^, Love Dalén^1,2,15*^

^1^ Centre for Palaeogenetics, Svante Arrhenius väg 20C, SE-106 91 Stockholm

^2^ Department of Bioinformatics and Genetics, Swedish Museum of Natural History, Stockholm, Sweden

^3^ Institute for Biochemistry and Biology, University of Potsdam, Karl-Liebknecht-Str. 24-25, 14476 Potsdam, Germany

^4^ Reiss-Engelhorn-Museen, Zeughaus C5, 68159 Mannheim, Germany

^5^ Academy of Sciences of Sakha Republic, Lenin Avenue 33, Republic of Sakha, Yakutia, Russia.

^6^ Museum "Severnyi Mir", Yakutsk, Russia

^7^ Mammoth Museum of Institute of Applied Ecology of the North, North-Eastern Federal University. Yakutsk, Republic Sakha (Yakutia), Russia

^8^ Institute of Plant and Animal Ecology, Russian Academy of Sciences, 202 Marta 8 St., 620144 Ekaterinburg, Russia

^9^ Department of Paleontology, University of Vienna, Althanstrasse 14, A-1090 Vienna, Austria

^10^ North-East Interdisciplinary Scientific Research Institute n.a. N.A. Shilo FEB RAS (NEISRI FEB RAS) 685000 Magadan, Portovaya Str.,16, Russia

^11^ Department of Earth Sciences, Natural History Museum, London, UK

^12^ Natural History Museum of Denmark, University of Copenhagen, Copenhagen, Denmark

^13^ OD Earth and History of Life, Royal Belgian Institute of Natural Sciences, Vautierstraat 29, 1000 Brussel, Belgium

^14^ The Francis Crick Institute, 1 Midland Road, London NW1 1AT

^15^ Department of Zoology, Stockholm University, Stockholm, Sweden

* Correspondence to: love.dalen@zoologi.su.se

^†^ Deceased, May 2020

**SUPPLEMENTARY METHODS**

**Identifying, describing and excluding *numt* sequence**

The *Panthera* lineage is known to have undergone the translocation of a large fragment from the mitochondria into the nuclear genome (*numt*, rather than cytoplasmic mitochondrial sequence *cymt*)^1,2^. In the single previous study that has used mitochondrial genomes from cave lions, this was dealt with by using a 2/3 consensus majority^2^, however this strategy may be ineffective leading to some incorrect base-calls^3^. We instead attempted to isolate the *numt* sequences using a modified version of the workflow described in Samaniego Castruita et al.^3^ (see Figure S1), whereby we phased the short reads mapped to the reference mitochondrial genome (above) and then concatenated our putative *cymt* and *numt* sequences and remapped to this as a consensus. This was done twice, so that the reads could also be mapped with the two regions in switched positions in the consensus to identify which of the phased consensus sequences was the *numt* and which was the *cymt* sequence. The effectiveness of this approach was determined based on a) the variation in pileup depth, and b) presence/absence of multi-allelic sites across the two concatenated sequences. A UPGMA phylogeny was constructed in Geneious 9.0.5 comparing the *Felis* *catus*, *P. tigris* and *P. spelaea* *numt* with the *F. catus*, *P. tigris*, *P. pardus*, *P. spelaea* and *P. leo* *cymt* (the *P. leo* and *P. pardus numts* are not available), with 1000 bootstrap replicates. The full mitochondrial sequence (15,280 bp), as well as the central *cymt* (6,317 bp; separately, phased using our pipeline) from one representative with high coverage was submitted to the MITOS web server (<http://mitos.bioinf.uni-leipzig.de/index.py>) for annotation. We did not attempt to annotate the *numt* sequence because all *numt* sequences recovered had regions with zero coverage (possibly an indication of deletions in the sequence, relative to the *cymt* sequence). While it was possible to separate the *numt* and *cymt* sequences for a subset of the high coverage mito-genomes (Supplementary Methods and Results), this was not the case for the majority of our samples and so for all analyses the *numt* region was excluded (unless explicitly stated otherwise, Figure S7), leaving a final trimmed sequence of 7,929 bp.


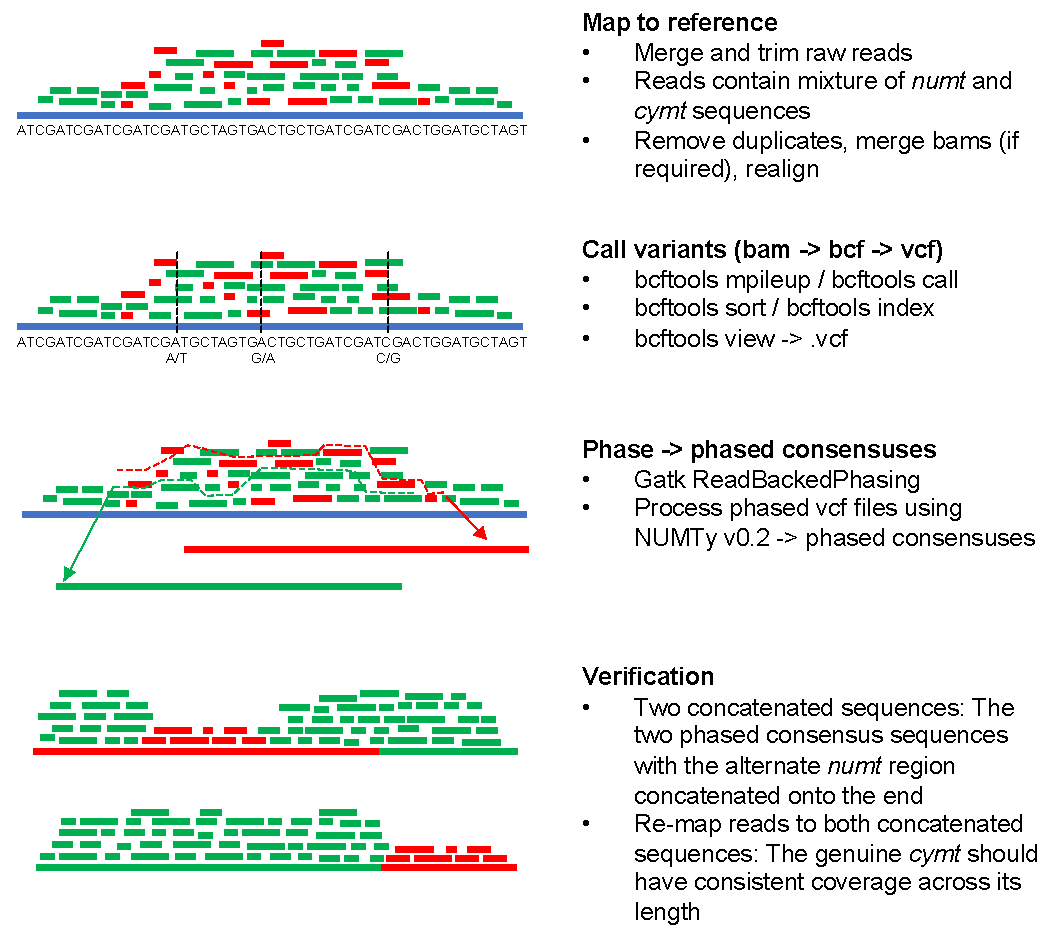


**Figure S1** *numt* identification pipeline. *Numt* sequence is shown in red and *cymt* is shown in green

**SUPPLEMENTARY RESULTS**

By combining newly generated ^14^C tip dates with split-time prior that takes into account fossil calibration, we estimate a split time between cave lions and lions of 1.85 mya (million radiocarbon years before present; 95% credibility interval: 0.52 – 2.91 mya). Using only fossil calibrations, the cave lion / lion divergence has previously been estimated at 2.08 mya^2^. This phylogeny recapitulates previous studies identifying reciprocally monophyletic clades in West/Central/North Africa and South Asia with respect to those from North East/South East/South West Africa (Figure 1; present study, 397 kya [95% credibility interval: 47.6 – 929 kya]; previous estimates, 245 kya^4^, 124 kya^5^). Using only ^14^C tip dates to inform the analysis leads to a divergence estimate that is considerably younger, at 550 kya (0.17 – 3.96 mya; Figure S2).

A randomization approach was carried out whereby ^14^C tip dates were randomized, with the hypothesis being that if the tip dates were providing little or no information into the tree, changing the tip dates would not significantly affect the posterior mutation rate^6^. This approach led to mutation rate estimates with 95%HPD intervals that did not overlap the tree mutation rate estimate (Figure S3), demonstrating that the tip dates we are using as priors are providing reliable mutation rate information into the trees, and thereby justifying our approach of including tip dates priors as part of the divergence time estimation.

Our results show clear evidence for a nuclear mitochondrial insertion, or *numt*, in our cave lion samples seen as variable sites and a plateau of higher than average coverage when reads were originally mapped to the cave lion reference sequence. We were able to extract this *numt* sequence from a subset of our samples with higher average coverage across the mitochondrial genome. The number of reads mapping to the *numt* were highly variable, and in some cases there may be more reads that map to the *numt* than the *cymt* portion of the mitochondrial sequence (e.g. Sample 10, Figure S6). This result strongly suggests that using a 2/3 consensus threshold is not an appropriate method^2^ and may lead to incorrect base calls or even bias the results towards the *numt* sequence rather than then *cymt* one. By mapping to a consensus that included both the putative *numt* and *cymt* regions, we were able to distinguish between the *numt* and *cymt* sequences. This is because the pileup depth of the reads was consistent across one of the consensus sequences (containing the *cymt*) and either higher or lower across the other (containing the *numt*, see Figure S6).

In the phylogeny comparing *F. catus*, *P. tigris* and *P. spelaea* *numt* sequences with the *F. catus*, *P. tigris*, *P. pardus*, *P. spelaea* and *P*. *leo* *cymt* sequences (Figure S7), the *spelaea numt* sequence clusters with the *tigris* *numt*, suggesting that the *numt* in cave lions shares a common ancestry with the one in other *Panthera* cats. However, the variation in size and position of the mitochondrial fragments that we observed between the cave lion *numt* and the other published felid *numts* (Figures S8 & S9) demonstrates that even if some of these *numt* sequences in different lineages originated from a common event, they have frequently undergone substantial evolutionary changes since that time.

**Figure S2** Phylogeny based on 7,929 bp of the mitochondrial genome with no prior used for the TMRCA of lions and cave lions. Branch thickness corresponds to posterior support, with exact values given on the nodes. Tip numbers correspond to sample IDs in Table S1, and posterior tip date estimates are given there. Scale on x-axis is in ^14^C years bp.


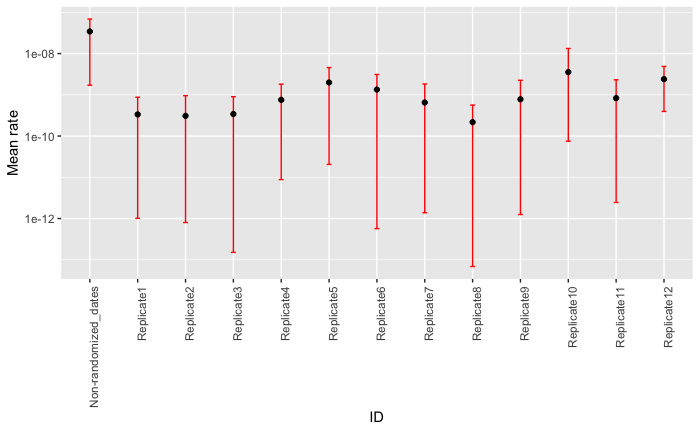


**Figure S3** Randomization approach to test the information content of the tip dates^6^. The mutation rate for the original date order is given by the left-most x-axis value.


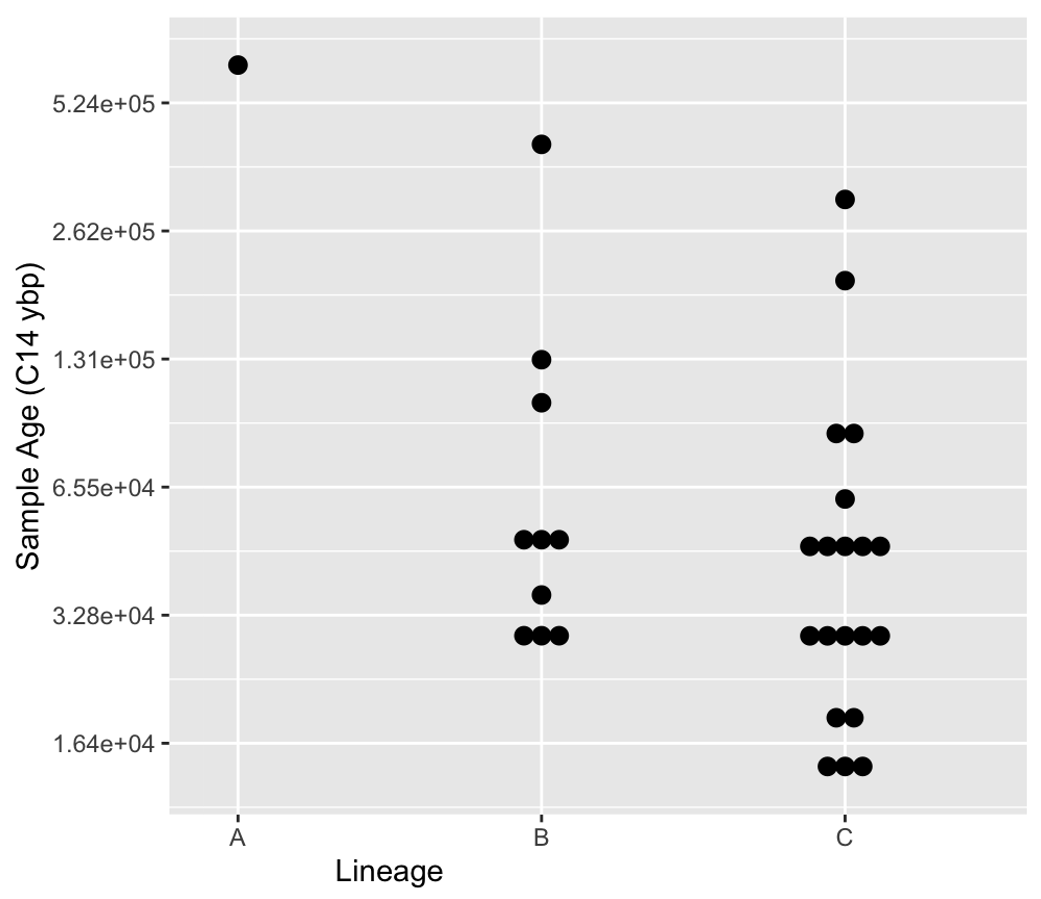


**Figure S4** Sample age versus lineage


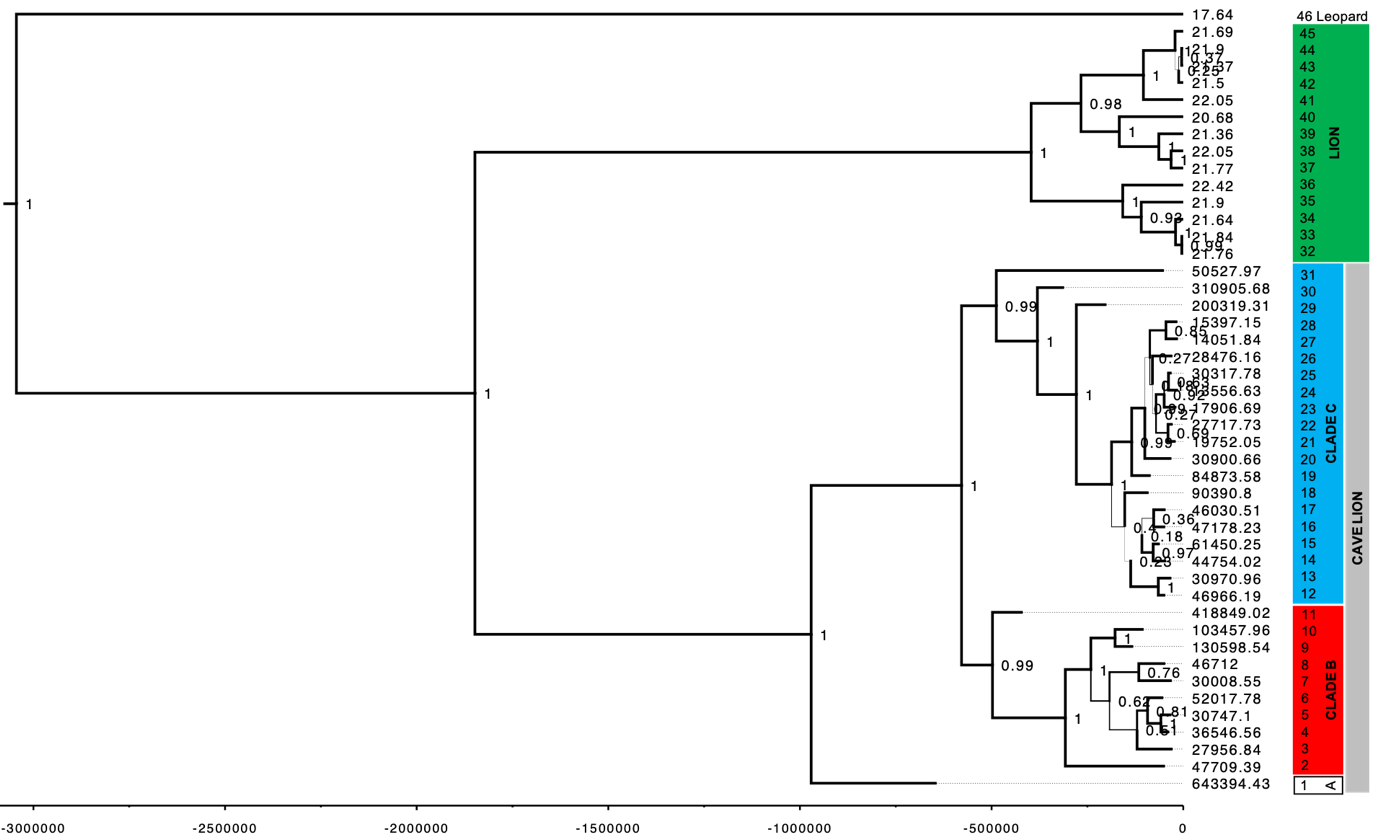


**Figure S5** Phylogeny based on 7,929 bp of the mitochondrial genome with median dates given on the tips. Priors used for the TMRCA between lions and cave lions were from Barnett et al.^2^ (2.08 mya, standard deviation of 0.52 mya). Branch thickness corresponds to posterior support, with exact values given on the nodes. Tip numbers in coloured boxes correspond to sample IDs in Table S1.

**
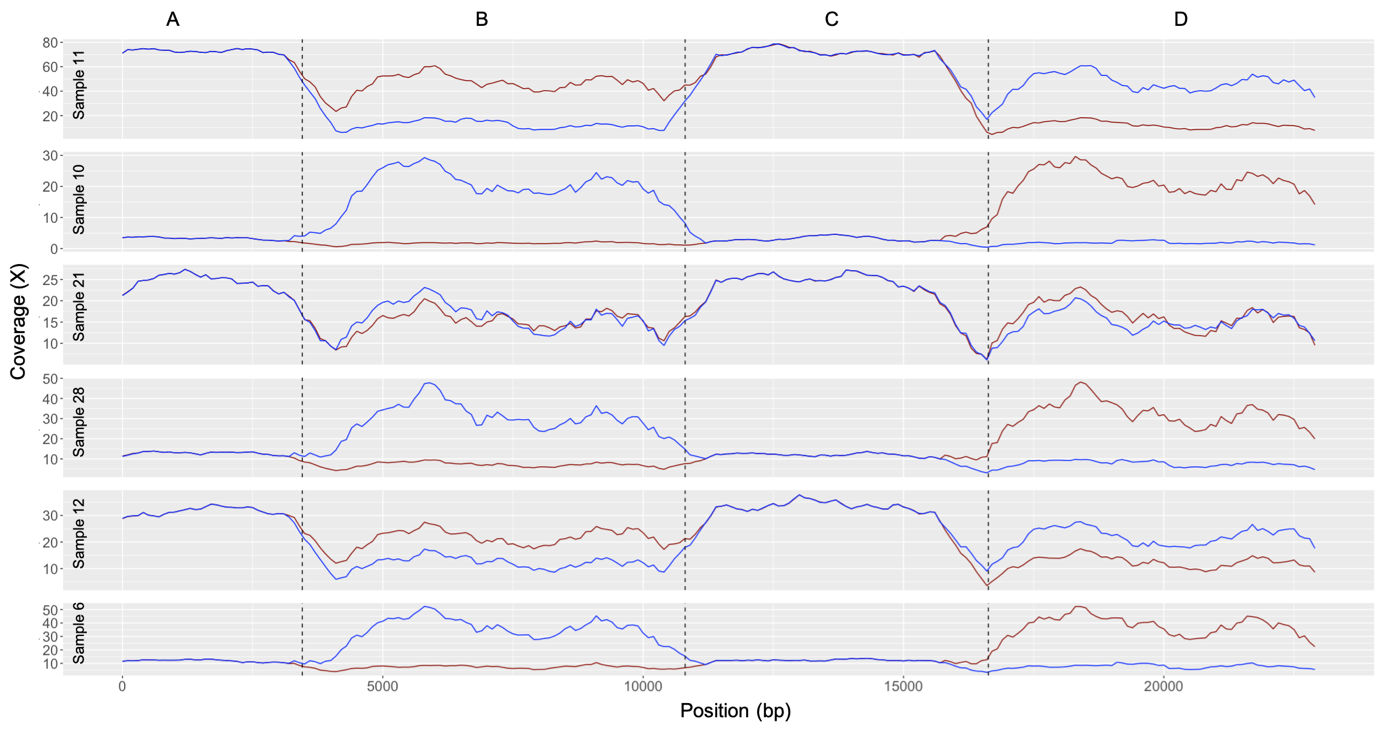
**

**Figure S6** Examples of coverage versus position (bp) of raw reads re-mapped onto the phased, concatenated *cymt* and *numt* consensuses (averaged over a 1000 bp sliding window in steps of 100 bp). This was done twice, with the regions in switched positions (red and blue lines). In most cases, the coverage within the putative *numt* region (black dashed lines, section B) was closer to the coverage in the *cymt* region (sections A and C) for one of the alternative concatenated consensuses (section D is the sequence concatenated onto the end), suggesting that that consensus contained the phased *cymt* sequence. Note that coverage over the *cymt* was not necessarily higher than over the *numt*, meaning that taking a consensus approach would lead to incorrect assignment of *cymt and numt* sequences (see e.g. the difference between samples 11 and 28). The phased *numt* sequence for sample 11 was used for annotation (Figure S3) and the UPGMA phylogeny (Figure S6).


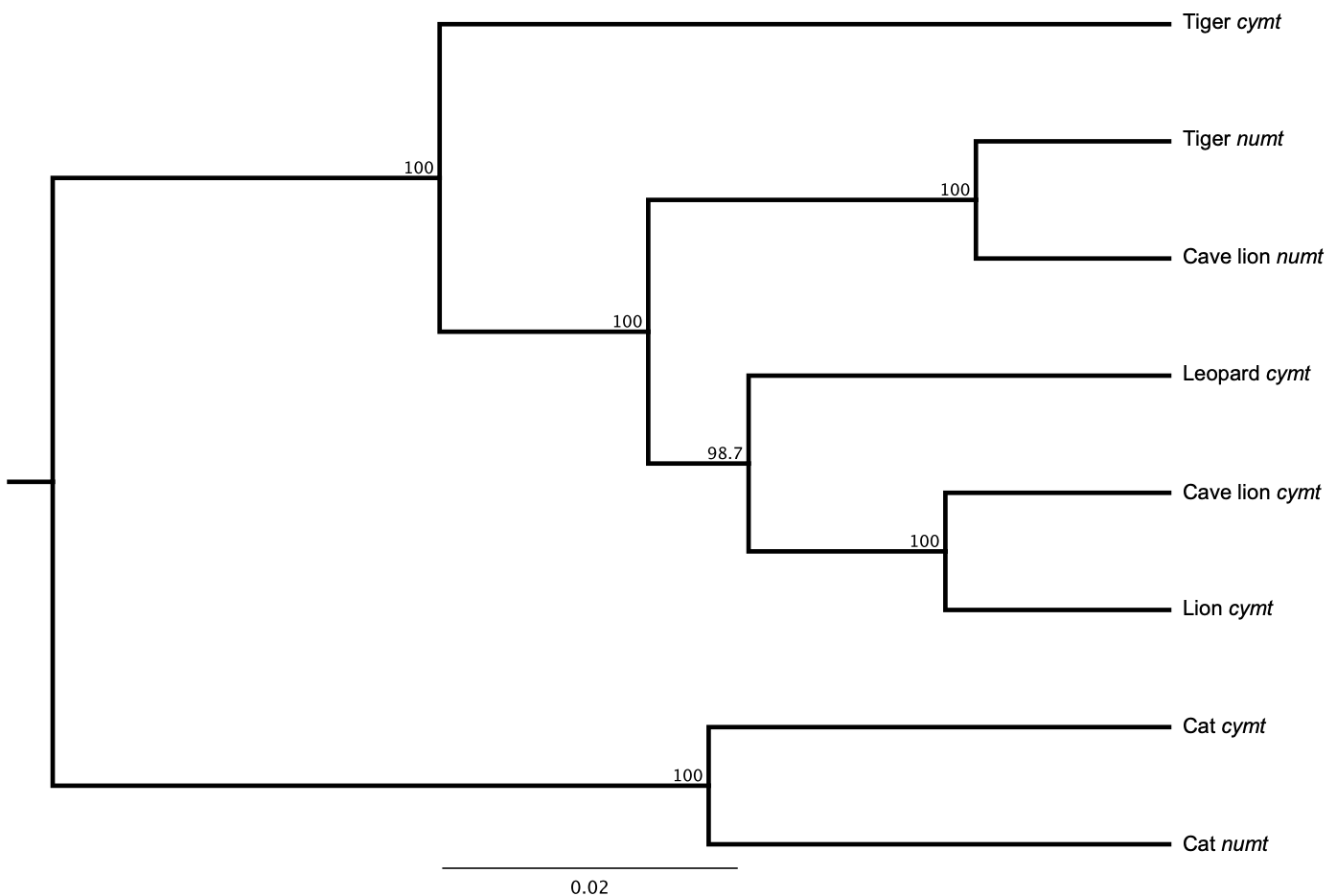


**Figure S7** UPGMA phylogeny of felid *cymts* and *numts* with 1000 bootstrap replicates.


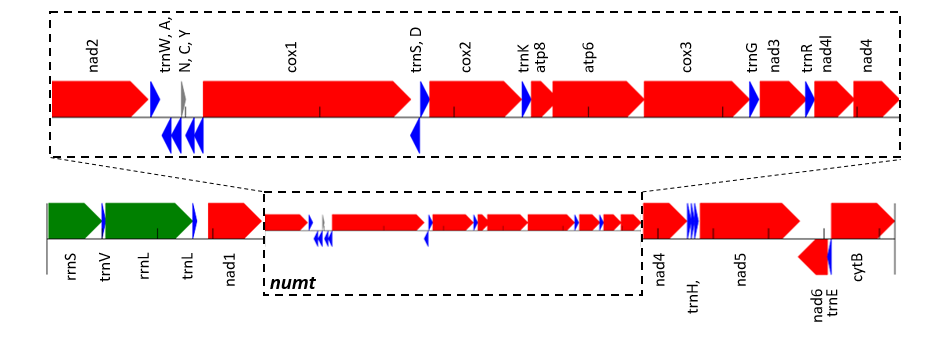


**Figure S8** The cave lion *numt*, the location of which was determined based on the presence of variable sites, and plateaus of coverage (Figure S5). Annotation was carried out on the MITOS web server (<http://mitos.bioinf.uni-leipzig.de/index.py>), using one representative with high coverage (“Sample 11”, Table S1). Protein coding genes are shown in red, ribosomal RNAs (rrn) in green and transfer RNAs (trn) in blue. The direction of the arrows show whether the feature is on the plus or minus strand (right plus, left minus; the thickness of the arrows is only for ease of visualisation). The region of the *numt* is indicated by the dashed box in the bottom panel, and shown in detail in the top panel.


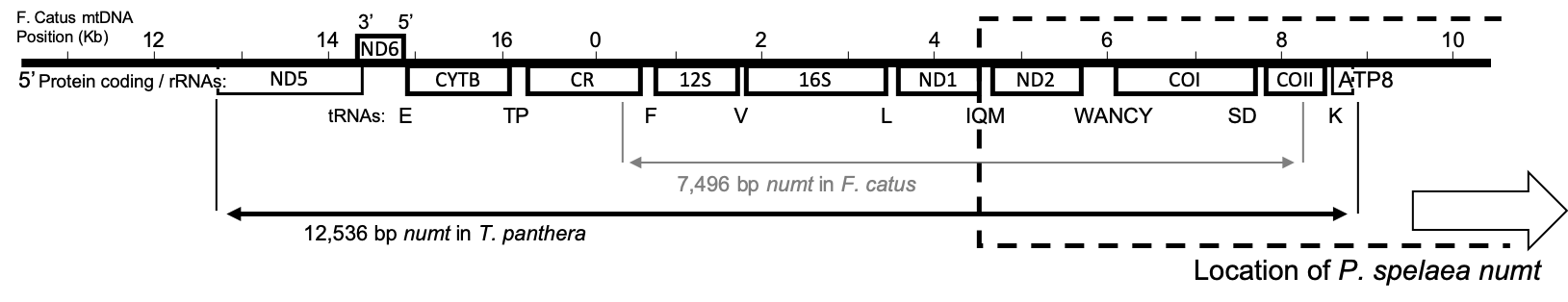


**Figure S9** Adapted figure from Kim et al.^1^ showing the location of the *P. spelaea numt* compared to the location of the *P. tigris* and *F. catus* ones reported in that study.

**Table S1** (Attached) Sample information including location, Genbank accession number and ^14^C date.

**Table S2** (Attached) Prior tip dates used and posterior tip dates recovered in BEAST analysis

**REFERENCES**

1. Kim, J. H. *et al.* Evolutionary analysis of a large mtDNA translocation (numt) into the nuclear genome of the *Panthera genus* species. *Gene* **366**, 292–302 (2006).

2. Barnett, R. *et al.* Mitogenomics of the extinct cave lion, *Panthera spelaea* (Goldfuss, 1810), resolve its position within the panthera cats. *Open Quat.* **2**, 1–11 (2016).

3. Samaniego Castruita, J. A., Zepeda Mendoza, M. L., Barnett, R., Wales, N. & Gilbert, M. T. P. Odintifier - A computational method for identifying insertions of organellar origin from modern and ancient high-throughput sequencing data based on haplotype phasing. *BMC Bioinformatics* **16**, 1–13 (2015).

4. Bertola, L. D. *et al.* Phylogeographic Patterns in Africa and High Resolution Delineation of Genetic Clades in the Lion (*Panthera leo*). *Sci. Rep.* **6**, 1–11 (2016).

5. Barnett, R. *et al.* Revealing the maternal demographic history of Panthera leo using ancient DNA and a spatially explicit genealogical analysis. *BMC Evol. Biol.* **14**, (2014).

6. Ho, S. Y. W. *et al.* Bayesian estimation of substitution rates from ancient DNA sequences with low information content. *Systematic Biology* **60**, 366–375 (2011).
